# Synthesis and Biological Investigation of some Novel Sulfonamide and Amide Derivatives Containing Coumarin Moieties 

**Published:** 2014

**Authors:** Mina Saeedi, Fereshteh Goli, Mohammad Mahdavi, Gholamreza Dehghan, Mohammad Ali Faramarzi, Alireza Foroumadi, Abbas Shafiee

**Affiliations:** a *Department of Medicinal Chemistry, Faculty of Pharmacy and Pharmaceutical Sciences Research Center, Tehran University of Medicinal Sciences, Tehran 14176, Iran. *; b *Department of Biology, Faculty of Natural Sciences, University of Tabriz, Iran. *; c *Department of Pharmaceutical Biotechnology, Faculty of Pharmacy, Tehran University of Medical Sciences, Tehran 14174, Iran. *

**Keywords:** Sulfonamides, Amides, Coumarins, Antioxidant activity, Antimicrobial

## Abstract

New sulfonamide and amide derivatives containing coumarin moieties; oxo-2H-chromen-sulfamoylphenylacetamides and oxo-2H-chromen-arylacetamides were synthesized starting from diverse 2-chloroacetamide derivatives and a wide range of coumarins. The structures of compounds were elucidated by IR and NMR spectra and also analytical elemental analysis. In the next step, the above mentioned compounds were screened for their antimicrobial and antioxidant activities.

Their antimicrobial activity was assigned using the conventional agar dilution method and the antioxidant activity was assessed using two methods, 1,1-diphenyl-2-picrylhydrazyl (DPPH) radical scavenging method and ferric reducing antioxidant power (FRAP) assay. Although the compounds showed no remarkable antimicrobial activities, most of them exhibited good antioxidant activities. Compounds 5b showed the most potent DPPH activity, whereas 8c was the most efficient compound in FRAP assay.

## Introduction

Sulfonamide and amide derivatives comprise an important class of drugs with diverse biological applications. Sulfonamides are widely used as antimicrobial (1, 2), anticancer (3, 4), anti-inflammatory (5) and antiviral agents as well as HIV protease inhibitors (6). Also some derivatives are well recognized as an antimetabolite (7) and in another study new sulfonamides have shown good cytotoxic effects against breast cancer cells (8). Sulfonamides were the first effective chemotherapeutic agents to be utilized efficiently to prevent and cure the bacterial infection in human beings (9-12). Recently some sulfonamide derivatives were screened for their antioxidant activity; the property which has not been investigated widely, and satosfactory results were obtained (13). 

Amides are one of the main building blocks in Nature (14) and have important biological activities (15, 16). Also they have been reported as antibiotic (17) and antimicrobial agents (18). Recent studies shows that novel designed amides which were active as novel orally available fatty acid amide hydrolase (FAAH) inhibitors are therapeutically effective against pain (19). Antimalaria (20), antiproliferative (21) and cyctotoxic activities (22) are other significant biological properties of amides. Valuable properties of sulfonamides and amides are true stimulation to present a large variety of these types of compounds and investigate their biological activity *in-vitro *and *in-vivo*. 

**Figure 1:**
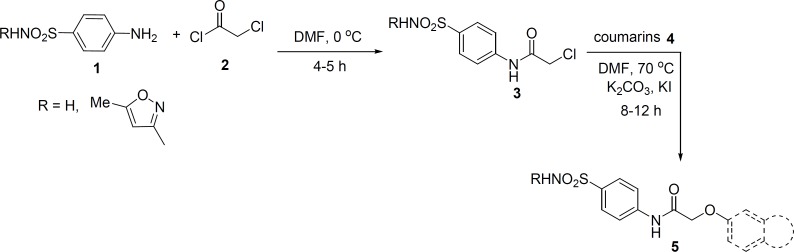
Synthesis of various sulfonamide derivatives 5.

**Figure 2:**
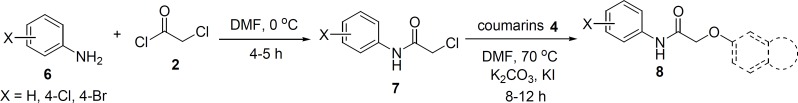
Synthesis of amide derivatives 8.

In continuation of our research program on the synthesis of bioactive molecules, we have investigated sulfonamides (23), amides (24, 25), and also coumarin derivatives (26-28) as they possess various precious biological activities (29- 31); in this report, we designed novel sulfonamide and amide derivatives containing coumarin moieties to profit both sufomide-coumarin/amide-coumarin properties (Figures 1 and 2). 

## Results and Discussion

Synthesis of sulfonamide derivatives 5 was started from sulfanilamide and its derivative, sulfamethoxazole 1 (Figure 1). They were reacted with chloroacetyl chloride 2, in DMF at 0 ºC to obtain 2-chloroacetamide derivatives 3. 

Then, we investigated reaction of 2-chloroacetamide derivatives 3 and various coumarin derivatives 4 under different conditions. It should be noted that the best reaction medium to obtain the desired compounds 5 was dimethylformamide (DMF). Also we found that using potassium carbonate (K_2_CO_3_) along with potassium iodide (KI) led to the formation of 5 in higher yields. 

It is worthwhile to mention that we prepared 3-chloroacetamide derivatives and examined their reaction with coumarin derivatives 4 under different conditions, no product or undesired products were obtained and further investigation was quitted. 

In the next step, as similar to the procedures for the synthesis of sulfonamides 5, amide derivatives 8 were prepared starting from anilines 6, chloroacetyl chloride 2, and coumarin derivatives 4 (Figure 2).

**Figure 3:**
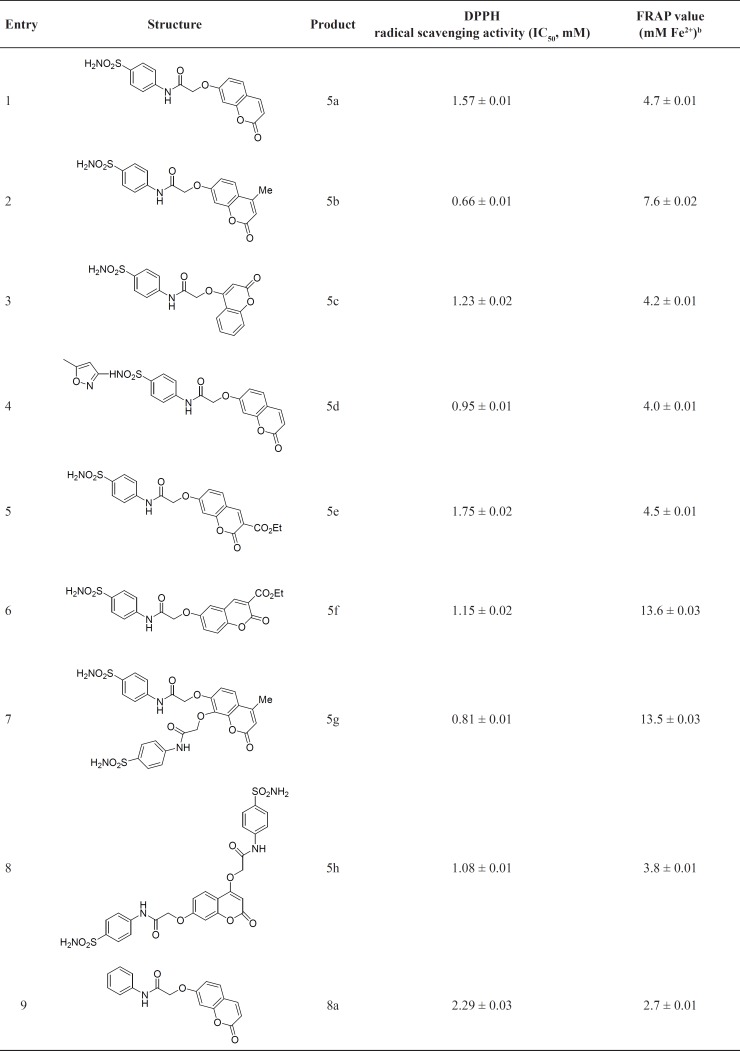
Coumarins used for the synthesis of sulfonamides 5 and amides 8

The structure of coumarin derivatives which were utilized for the synthesis of compounds 5 and 8 have been depicted in Figure 3. They include 7-hydroxycoumarin 4a, 7-hydroxy-4-methylcoumarin 4b, 7-hydroxy- 4-phenylcoumarin 4c, 4-hydroxycoumarin 4d, 4,7-dihydroxycoumarin 4e, 7,8-dihydroxy-4- methylcoumarin 4f, ethyl-7-hydroxycoumarin-3-carboxylate 4g, and ethyl-6-hydroxycoumarin- 3-carboxylate 4h. The corresponding products are shown in Table 1 (Entries 1-18). 

**Table 1:** Sulfonamide and amide derivatives and their antioxidant activities

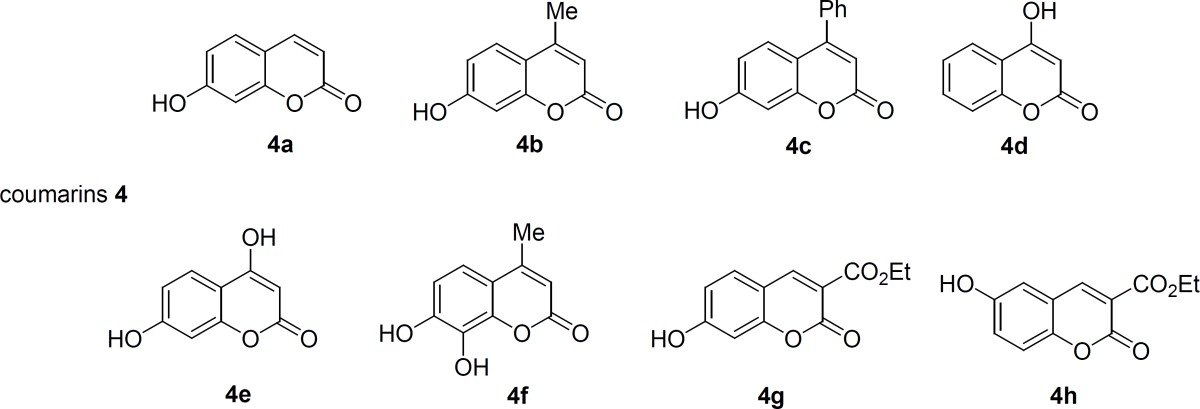

^a^In μM

^b^100 μg of substances were used.

*Antimicrobial activity*

Compounds 5a-h and 8a-j were evaluated for their antibacterial activity against Gram-positive (*Staphylococcus aureus *ATCC 6538p, *Staphylococcus epidermidis *ATCC 12228) and Gram-negative (*Escherichia coli *ATCC 8739, *Klebsiella pneumonia *ATCC 10031, and *Pseudomonas aeruginosa *ATCC 9027) bacteria using conventional agar-dilution method (32). The MIC (minimum inhibitory concentration) values were determined by comparison to ciprofloxacin as a reference drug. None of the compounds showed antimicrobial activities below 100 μM. 

*Antioxidant activity*

In this study, antioxidant activity of compounds 5a-h and 8a-j were assessed using two different assay systems; DPPH (1,1-diphenyl-2- picrylhydrazyl) (33) and FRAP (ferric reducing antioxidant power) (34). 

*DPPH radical scavenging activity*

The free radical scavenging activity of sulfonamide and amide derivatives 5a-h and 8a-j was evaluated by DPPH colorimetric method (33). Several dilutions of compounds were made and assayed to obtain concentration of the sample required to scavenge 50% (IC_50_) of DPPH-free radical applying suitable regression analysis of the mean values. The results are given in Table 1. 

All sulfonamide derivatives exhibited moderate radical scavenging activities (0.66 to 1.75 mM). The results indicate that various factors such as nature of substituents at position 3 or 4 and also position of hydroxyl group in the utilized coumarins 4 are very important and affect the related radical scavenging activity. Obviously presence of methyl group in the sulfonamide derivatives has significant effect since the best result is related to 5b (0.66 mM) which possess methyl group at position 4. Also this effect is clear in compound 5g (0.81 mM) which showed good activity, but introduction of two oxy-*N*-(4-sulfamoylphenyl)acetamide groups reduced the activity in comparison to 5b. It should be noted that the presence of ethyl carboxylate group at position 3 diminished radical scavenging activity as well. Another noticeable factor is position of hydroxyl group in coumarins 4. According to the results in Table 1, the product derived from 4-hydroxycoumarin 5c (1.23 mM) is more active than the product derived from 7-hydroxycoumarin 5a (1.57 mM). It should be noted that introduction of methoxazole ring in sulfonamide moiety, 5d (0.95 mM) is effective and it has improved the activity of 5d in comparison to 5a (1.57 mM). 

Similar results can be seen in the case of amides. Amides derived from 4-hydroxycoumarin 8d (1.11 mM) and 8e (1.23 mM) are more active than compounds derived from 7-hydroxycoumarin 8a (2.29 mM) and 8b (1.14 mM). Also compounds containing phenyl group at position 4, 8i and 8j show better activity in comparison to those of having methyl group at the same position (8f-h). Another factor is related to the presence of halogen atom on the aromatic ring. We found that 4-bromophenyl enhanced the radical scavenging activity. In this manner, the best result is related to 8c (0.94 mM).

To sum up, considering the above mentioned factors, better radical scavenging activities are related to compounds 5b, 5g, 8c and 8j. 

*FRAP ability*

The FRAP assay measures the ability of a compound to reduce the ferric 2,4,6-tripyridyl-s-triazine complex to the colored ferrous complex (34). FRAP values are obtained by comparing the absorbance change at 593 nm in test reaction mixtures with those containing ferrous ions in known concentration. The FRAP values for the tested compounds (Table 1) revealed that the synthesized compounds showed noticeable activities. 

Among sulfonamides, 5f and 5g; compounds derived from 7-hdroxycoumarin and 7,8-dihydroxycoumarin, showed the best results (13.6 and 13.5 mM, respectively). Contrary to DPPH radical scavenging activities, compound derived from 7-hdroxycoumarin 5a (4.7 mM) exhibited better result in comparison to compound derived from 4-hdroxycoumarin 5c (4.2 mM) and also presence of ethyl carboxylate group at position 3 increased the activity; see related data for compounds 5e and 5f in Table 1. Similar to DPPH results, compounds containing methyl group at position 4, 5b and 5g showed satisfactory results (7.6 and 13.5 respectively).

As can be seen in Table 1, amides exhibited better FRAP values and similar to DPPH results amides containing methyl at position 4 and 4-bromophenyl in their structure showed very good results (8f and 8c; 15.4 and 19.1 mM, respectively). There are two significant data in Table 1, related to 8d and 8e with values of 17.5 and 17.3 mM, respectively. These compounds have been derived from 4-hdroxycoumarin and are more active in comparison to 8a and 8b which were derived from 7-hdroxycoumarin. It is worthwhile to remind that introduction of bromine in the structure of the synthesized amides showed remarkable effect and in brief, according to Table 1, the best result is related to 8c which involves 4-bromophenyl in the structure and this is in good agreement with the results from DPPH radical scavenging activity test. 

## Experimental

*General*

Melting points were taken on a *Kofler *hot stage apparatus and are uncorrected. ^1^H and ^13^C NMR spectra were recorded on *Bruker FT-500*, using TMS as an internal standard. The IR spectra were obtained on a *Nicolet Magna FTIR 550 *spectrophotometer (in KBr). 

*Chemicals*

All chemicals, reagents and solvents were obtained from Sigma-Aldrich and Merck chemical companies and were used without any purification. Some coumarins were prepared according to references 35 and 36. TLC analyses were performed on a 3-10 cm aluminum sheet pre-coated with silica gel 60-254 (Merck). Visualization of spots on TLC plate was accomplished with UV light. 

*General procedure for synthesis of sulfonamides 5 and amides 8*

*Synthesis of 2-chloroacetamide derivatives 3 and* ***7***

Chloroacetylchloride 2 (40 mmol) was added to a solution of sulfanilamide 5 or aniline 6 derivatives (20 mmol) in DMF (20 mL) at 0 ◦C, and the reaction was continued at room temperature for 5 h. After completion of reaction, the mixture was poured in cold water and the precipitate was filtered off and the pure products were obtained by recrystallization from H_2_O or H_2_O/EtOH. 

*Synthesis of sulfonamide and amide derivatives 5 and 8*

To a solution of 2-chloroacetamide derivatives 3 or 7 (2 mmol) in DMF (20 mL), coumarin derivatives 4 (2 mmol), potassium carbonate K_2_CO_3_ (2 mmol), and potassium iodide KI (2 mmol) were added and the reaction mixture was heated at 70 ◦C for 8-12 h. After completion of the reaction, the mixture was poured in cold water and the precipitate was filtered off and the pure products were obtained by recrystallization from EtOH. 

*2-((2-Oxo-2H-chromen-7-yl)oxy)-N-(4-sulfamoylphenyl)acetamide (5a)*

Yield: 75 %; mp 266-268 ◦C; IR υ_max_ (KBr): 3390, 3341, 3250, 3094, 2980, 1723, 1622, 1530, 1404, 1329, 1155; ^1^H NMR (500 MHz, DMSO-*d*_6_): 10.50 (s, NH), 8.00 (d, *J *= 8.5 Hz, 1H, H_4_), 7.80-7.78 (m, 4H, ArH), 7.58 (d, *J *= 8.0 Hz, 1H, H_5_), 7.28 (s, 2H, NH_2_), 7.07-7.04 (m, 2H, H_6_, H_8_), 6.32 (d, *J *= 8.5 Hz, 1H, H_3_), 4.90 (s, 2H, OCH_2_); ^13^C NMR (125 MHz, DMSO-*d*_6_): 166.5, 160.9, 160.2, 155.2, 144.2, 141.2, 138.9, 129.6, 127.7, 126.7, 119.2, 112.9, 112.7, 101.7, 67.2. Anal. Calcd for C_17_H_14_N_2_O_6_S: C, 54.54; H, 3.77; N, 7.48. Found: C, 54.27; H, 3.50; N, 7.75.

*2-((4-Methyl-2-oxo-2H-chromen-7-yl)oxy)-N-(4-sulfamoylphenyl)acetamide (5b)*

Yield: 70 %; mp 295-298 ◦C; IR υ_max_ (KBr): 3308, 3262, 3190, 3121, 1698, 1612, 1540, 1392, 1332, 1159; ^1^H NMR (500 MHz, DMSO-*d*_6_): 10.51 (s, 1H, NH), 7.82-7.79 (m, 4H, ArH), 7.72 (d, *J *= 8.6 Hz, 1H, H_5_), 7.30 (s, 2H, NH_2_), 7.07-7.04 (m, 2H, H_6_, H_8_), 6.23 (s, 1H, H_3_), 4.91 (s, 2H, OCH_2_), 2.40 (s, 3H, CH_3_); ^13^C NMR (125 MHz, DMSO-*d*_6_): 166.5, 160.8, 160.1, 154.5, 153.4, 141.2, 138.9, 126.7, 126.6, 119.2, 113.7, 112.4, 111.5, 101.7, 67.2, 18.1. Anal. Calcd for C_18_H_16_N_2_O_6_S: C, 55.66; H, 4.15; N, 7.21. Found: C, 55.38; H, 4.32; N, 7.50.

*2-((2-Oxo-2H-chromen-4-yl)oxy)-N-(4-sulfamoylphenyl)acetamide (5c)*

Yield: 77 %; mp 164-165 ◦C; IR υ_max_ (KBr): 3470, 3316, 3120, 3094, 1692, 1618, 1541, 1496, 1335, 1161; ^1^H NMR (500 MHz, DMSO-*d*_6_): 10.61 (s, 1H, NH), 7.95 (d, *J *= 8.5 Hz, 1H, H_5_), 7.80-7.78 (m, 4H, ArH), 7.70 (t, *J *= 8.0 Hz, 1H, H_7_), 7.44-7.42 (m, 2H, H_6_, H_8_), 7.30 (s, H, NH_2_), 5.93 (s, 1H, H_3_), 5.08 (s, 2H, OCH_2_); ^13^C NMR (125 MHz, DMSO-*d*_6_): 165.1, 164.5, 161.4, 152.7, 141.1, 138.9, 132.9, 126.7, 124.3, 123.2, 119.2, 116.4, 115.0, 91.3, 67.5. Anal. Calcd for C_17_H_14_N_2_O_6_S: C, 54.54; H, 3.77; N, 7.48. Found: C, 54.80; H, 3.90; N, 7.15. 

*N-(4-(N-(5-Methylisoxazol-3-yl)sulfamoyl)phenyl)-2-((2-oxo-2H-chromen-7-yl)oxy)acetamide (5d)*

Yield: 65 %; mp 274-277 ◦C; IR υ_max_ (KBr): 3457, 3348, 3088, 2922, 1732, 1626, 1531, 1464, 1301, 1134; ^1^H NMR (500 MHz, DMSO-*d*_6_): 10.30 (s, 1H, NH), 8.00 (d, *J *= 8.8 Hz, 1H, H_4_), 7.66 (d, *J *= 8.0 Hz, 1H, H_5_), 7.61-7.59 (m, 4H, ArH), 7.05-7.03 (m, 2H, H_6_, H_8_), 6.31 (d, *J *= 8.8 Hz, 1H, H_3_), 5.74 (s, 1H, methoxazole), 4.85 (s, 2H, OCH_2_), 2.09 (s, 3H, CH_3_); ^13^C NMR (125 MHz, DMSO-*d*_6_): 166.1, 165.9, 165.5, 160.9, 160.2, 155.1, 144.2, 142.8, 138.9, 129.5, 126.7, 124.9, 118.7, 112.9, 112.6, 101.7, 97.0, 67.2, 12.2. Anal. Calcd for C_21_H_17_N_3_O_7_S: C, 55.38; H, 3.76; N, 9.23. Found: C, 55.19; H, 3.92; N, 9.44.

*Ethyl2-oxo-7-(2-oxo-2-((4-sulfamoylphenyl)amino)ethoxy)-2H-chromen-3-carboxylate (5e)*

Yield: 60 %; mp 295-298 ◦C; IR υ_max_ (KBr): 3420, 3335, 3251, 3088, 2983, 1755, 1709, 1611, 1528, 1434, 1377, 1157; ^1^H NMR (500 MHz, DMSO-*d*_6_): 10.53 (s, 1H, NH), 8.74 (s, 1H, H_4_), 7.89 (d, *J *= 8.5 Hz, H_5_), 7.79-7.74 (m, 4H, ArH), 7.28 (s, 2H, NH_2_), 7.11-7.08 (m, 2H, H_6_, H_8_), 4.96 (s, 2H, OCH_2_), 4.30 (q, *J *= 7.0 Hz, 2H, OCH_2_CH_3_), 1.31 (t, *J *= 7.0 Hz, 3H, OCH_2_CH_3_); ^13^C NMR (125 MHz, DMSO-*d*_6_): 166.6, 163.6, 157.1, 156.6, 149.5, 141.6, 139.3, 132.1, 127.2, 119.6, 118.7, 114.2, 114.0, 113.0, 112.4, 67.7, 61.4, 14.5. Anal. Calcd for C_20_H_18_N_2_O_8_S: C, 53.81; H, 4.06; N, 6.27. Found: C, 53.98; H, 3.90; N, 6.41.

*Ethyl2-oxo-6-(2-oxo-2-((4-sulfamoylphenyl)amino)ethoxy)-2H-chromen-3 carboxylate (5f)*

Yield: 65 %; mp 266-268 ◦C; IR υ_max_ (KBr): 3354, 3251, 1750, 1692, 1572, 1535, 1491, 1320, 1170; ^1^H NMR (500 MHz, DMSO-*d*_6_): 10.48 (s, 1H, NH), 8.72 (s, 1H, H_4_), 7.85-7.83 (m, 4H, ArH), 7.54 (d, *J *= 8.0 Hz, 1H, H_7_), 7.56-7.52 (m, 2H, H_5_, H_8_), 7.28 (s, 2H, NH_2_), 4.83 (s, 2H, OCH_2_), 4.30 (q, *J *= 6.9 Hz, 2H, OCH_2_CH_3_), 1.31 (t, *J *= 6.9 Hz, 3H, OCH_2_CH_3_); ^13^C NMR (125 MHz, DMSO-*d*_6_): 166.7, 162.6, 156.1, 154.2, 149.3, 148.3, 141.2, 138.8, 126.7, 122.7, 119.2, 118.1, 118.0, 117.3, 113.1, 67.4, 61.2, 14.0. Anal. Calcd for C_20_H_18_N_2_O_8_S: C, 53.81; H, 4.06; N, 6.27. Found: C, 53.52; H, 4.22; N, 6.01.

*2-[(4-Methyl-2-oxo-8-{[(4-sulfamoylphenyl)carbamoyl]methoxy}-2H-chromen-7-yl)oxy]-N-* *(4-sulfamoylphenyl)acetamide (5g)*

Yield: 80 %; mp> 300 ◦C; IR υ_max _(KBr): 3345, 3255, 3120, 2920, 1695, 1615, 1530, 1405, 1350, 1148; ^1^H NMR (500 MHz, DMSO-*d*_6_): 10.50 (s, 1H, NH), 11.10 (s, 1H, NH), 7.90-7.69 (m, 8H, ArH), 7.53 (d, *J *= 9.0 Hz, 1H, H_5_), 7.29 (s, 4H, 2NH_2_), 7.17 (d, *J *= 9.0 Hz, 1H, H_6_), 6.30 (s, 1H, H_3_), 5.10 (s, 2H, OCH_2_), 4.88 (s, 2H, OCH_2_), 2.41 (s, 3H, CH_3_); ^13^C NMR (125 MHz, DMSO-*d*_6_): 167.9, 166.0, 159.9, 154.1, 153.3, 147.1, 141.5, 139.1, 133.8, 132.6, 129.9, 127.4, 127.0, 121.2, 119.7, 119.4, 115.1, 112.2, 110.28, 72.2, 68.0, 18.7; Anal. Calcd for C_26_H_24_N_4_O_10_S_2_: C, 50.64; H, 3.92; N, 9.09. Found: C, 50.83; H, 3.74; N, 9.32. 

*2-[(2-Oxo-4-{[(4-sulfamoylphenyl)carbamoyl]methoxy}-2H-chromen-7-yl)oxy]-N-(4-sulfampylphenyl)acetamide (5h)*

Yield: 80 %; mp 205-207 ◦C; IR υ_max_ (KBr): 3278, 3127, 1691, 1616, 1535, 1405, 1326, 1155; ^1^H NMR (500 MHz, DMSO-*d*_6_): 10.61 (s, 1H, NH), 10.53 (s, 1H, NH), 7.89 (d, *J *= 8.6 Hz, 1H, H_5_), 7.83-7.76 (m, 8H, ArH), 7.30 (s, 4H, 2NH_2_), 7.12-7.08 (m, 2H, H_6_, H_8_), 5.79 (s, 1H, H_3_), 5.06 (s, 2H, OCH_2_), 4.93 (s, 2H, OCH_2_); ^13^C NMR (125 MHz, DMSO-*d*_6_): 166.4, 165.2, 164.9, 161.8, 161.5, 154.4, 141.2, 141.1, 139.0, 138.9, 127.8, 126.8, 126.7, 124.5, 119.3, 112.6, 108.7, 101.5, 89.0, 67.5, 67.2. Anal. Calcd for C_25_H_22_N_4_O_10_S_2_: C, 49.83; H, 3.68; N, 9.30. Found: C, 49.59; H, 3.49; N, 9.51.

*2-(2-Oxo-2H-chromen-7-yloxy)-N-phenylacetamide (8a)*

Yield: 75 %; mp 195-196 ◦C; IR υ_max_ (KBr): 3375, 3057, 1725, 1675, 1594, 1530; ^1^H NMR (500 MHz, DMSO-*d*_6_): 10.17 (s, 1H, NH), 7.99 (d, *J *= 9.5 Hz, 1H, H_4_), 7.66 (d, *J *= 8.4 Hz, 1H, H_5_), 7.63 (d, *J *= 7.7 Hz, 2H, H_2_›, H_6_›), 7.33 (t, *J *= 7.7 Hz, 2H, H_3_›, H_5_›), 7.10 (t, *J *= 7.7 Hz, 1H, H_4_›), 7.07-7.03 (m, 2H, H_6_, H_8_), 6.30 (d, *J *= 9.5 Hz, 1H, H_3_), 4.85 (s, 2H, OCH_2_); ^13^C NMR (125 MHz, DMSO-*d*_6_): 165.9, 161.0, 160.3, 155.2, 144.3, 138.3, 129.6, 128.8, 123.9, 119.7, 113.0, 112.9, 112.8, 101.7, 67.3. Anal. Calcd for C_17_H_13_NO_4_: C, 69.15; H, 4.44; N, 4.74. Found: C, 68.88; H, 4.28; N, 4.96.

*2-(2-Oxo-2H-chromen-7-yloxy)-N-(4-chlorophenyl)-acetamide (8b)*

Yield: 70 %; mp 226-228 ◦C; IR υ_max_ (KBr): 3357, 1701, 1624, 1544, 1493; ^1^H NMR (500 MHz, DMSO-*d*_6_): 10.31 (s, 1H, NH), 7.99 (d, *J *= 9.5 Hz, 1H, H_4_), 7.67-7.65 (m, 3H, H_5_, H_2_›, H_6_›), 7.38 (t, *J *= 8.8 Hz, 2H, H_3_›, H_5_›), 7.04-7.02 (m, 2H, H_6_, H_8_), 6.30 (d, *J *= 9.5 Hz, 1H, H_3_), 4.84 (s, 2H, OCH_2_); ^13^C NMR (125 MHz, DMSO-*d*_6_): 166.1, 160.9, 160.3, 155.2, 144.3, 137.3, 129.6, 128.8, 127.4, 121.3, 112.9, 112.8, 101.7, 67.3. Anal. Calcd for C_17_H_12_ClNO_4_: C, 61.92; H, 3.67; N, 4.25. Found: C, 62.15; H, 3.83; N, 4.02.

*2-(2-Oxo-2H-chromen-7-yloxy)-N-(4-bromophenyl)-acetamide (8c)*

Yield: 70 %; mp 205-208 ◦C; IR υ_max_ (KBr): 3356, 3093, 1732, 1683, 1627, 1590, 1540, 1492; ^1^H NMR (500 MHz, DMSO-*d*_6_): 10.31 (s, 1H, NH), 8.00 (d, *J *= 9.4 Hz, 1H, H_4_), 7.67 (d, *J *= 9.1 Hz, 1H, H_5_), 7.61 (d, *J *= 8.8 Hz, 2H, H_2_›, H_6_›), 7.52 (t, *J *= 8.8 Hz, 2H, H_3_›, H_5_›), 7.05-7.03 (m, 2H, H6, H8), 6.31 (d, *J *= 9.4 Hz, 1H, H_3_), 4.85 (s, 2H, OCH_2_); ^13^C NMR (125 MHz, DMSO-*d*_6_): 166.1, 160.9, 160.2, 155.2, 144.3, 137.7, 131.6, 129.6, 121.7, 115.5, 112.9, 112.8, 101.7, 67.3. Anal. Calcd for C_17_H_12_BrNO_4_: C, 54.57; H, 3.23; N, 3.74. Found: C, 54.23; H, 2.98; N, 3.28.

*2-(2-Oxo-2H-chromen-4-yloxy)-N-phenylacetamide (8d)*

Yield: 70 %; mp 251-253 ◦C; IR υ_max _(KBr): 3360, 3283, 3139, 1700, 1680, 1618, 1567, 1495; ^1^H NMR (500 MHz, DMSO-*d*_6_): 10.29 (s, 1H, NH), 7.95 (d, *J *= 7.8 Hz, 1H, H_5_), 7.70 (t, *J *= 7.8 Hz, H_7_), 7.62 (d, *J *= 7.5 Hz, H_2_›, H_6_›), 7.45-7.40 (m, 2H, H_6_, H_8_), 7.35 (t, *J *= 7.5 Hz, H_3_›, H_5_›), 7.11 (t, *J *= 7.5 Hz, H_4_›), 5.90 (s, 1H, H_3_), 5.04 (s, 2H, OCH_2_); ^13^C NMR (125 MHz, DMSO-*d*_6_): 164.6, 164.5, 161.5, 152.8, 138.2, 133.0, 128.9, 124.3, 123.9, 123.3, 119.6, 116.5, 115.0, 91.2, 67.7; Anal. Calcd for C_17_H_13_NO_4_: C, 69.15; H, 4.44; N, 4.74. Found: C, 69.30; H, 4.73; N, 4.90.

*2-(2-Oxo-2H-chromen-4-yloxy)-N-(4-chlorophenyl)acetamide (8e)*

Yield: 70 %; mp 260-262 ◦C; IR υ_max_ (KBr): 3421, 3288, 3139, 1700, 1685, 1618, 1490; ^1^H NMR (500 MHz, DMSO-*d*_6_): 10.41 (s, 1H, NH), 7.95 (d, *J *= 8.9 Hz, 1H, H_5_), 7.45-7.40 (m, 4H, H_2’_, H_3_’, H_5’_, H_6’_), 7.70 (m, 3H, H_6_, H_7_, H_8_), 5.91 (s, 1H, H_3_), 5.03 (s, 2H, OCH_2_); ^13^C NMR (125 MHz, DMSO-*d*_6_): 165.1, 165.0, 161.9, 153.2, 137.6, 133.4, 129.2, 127.9, 124.7, 123.7, 121.7, 116.9, 115.4, 91.7, 68.0; Anal. Calcd for C_17_H_12_ClNO_4_: C, 61.92; H, 3.67; N, 4.25. Found: C, 62.09; H, 3.91; N, 4.05.

*2-(4-Methyl-2-oxo-2H-chromen-7-yloxy)-N-phenylacetamide (8f)*

Yield: 72 %; mp 232-234 ◦C; IR υ_max_ (KBr): 3365, 1700, 1680, 1629, 1541, 1501, 1393; ^1^H NMR (500 MHz, DMSO-*d*_6_): 10.18 (s, 1H, NH), 7.72 (d, *J *= 8.7 Hz, 1H, H_5_), 7.64 (d, *J *= 7.8 Hz, 2H, H_2›_, H_6›_), 7.33 (t, *J *= 7.8 Hz, 1H, H_3›_, H_5›_), 7.11-7.03 (m, 3H, H_6_, H_8_, H_4›_), 6.21 (s, 1H, H_3_), 4.85 (s, 2H, OCH_2_), 2.40 (s, 3H, CH_3_); ^13^C NMR (125 MHz, DMSO-*d*_6_): 165.8, 160.8, 160.1, 154.5, 153.4, 138.3, 128.8, 126.6, 123.8, 119.7, 113.6, 112.4, 111.4, 101.7, 67.3, 18.1; Anal. Calcd for C_18_H_15_NO_4_: C, 69.89; H, 4.89; N, 4.53. Found: C, 70.05; H, 4.56; N, 4.38.

*2-(4-Methyl-2-oxo-2H-chromen-7-yloxy)-N-(4-chlorophenyl)acetamide (8g)*

Yield: 75 %; mp 238-240 ◦C; IR υ_max_ (KBr): 3359, 2914, 1705, 1680, 1593, 1536, 1490; ^1^H NMR (500 MHz, DMSO-*d*_6_): 10.31 (s, 1H, NH), 7.73 (d, *J *= 8.7 Hz, 1H, H_5_), 7.67 (d, *J *= 8.7 Hz, 1H, H_2›_, H_6›_), 7.38 (t, *J *= 8.7 Hz, 1H, H_3›_, H_5›_), 7.07-7.03 (m, 2H, H_6_, H_8_), 6.24 (s, 1H, H_3_), 4.86 (s, 2H, OCH_2_), 2.41 (s, 3H, CH_3_); ^13^C NMR (125 MHz, DMSO-*d*_6_): 166.5, 161.2, 160.5, 154.9, 153.8, 137.7, 129.1, 127.8, 127.0, 121.6, 114.1, 112.8, 111.9, 102.1, 67.6, 18.6; Anal. Calcd for C_18_H_14_ClNO_4_: C, 62.89; H, 4.10; N, 4.07. Found: C, 62.63; H, 3.88; N, 4.25.

*2-(4-Methyl-2-oxo-2H-chromen-7-yloxy)-N-(4-bromophenyl)acetamide (8h)*

Yield: 68 %; mp 259-262 ◦C; IR υ_max_ (KBr): 3360, 2913, 1705, 1680, 1623, 1532, 1490; ^1^H NMR (500 MHz, DMSO-*d*_6_): 10.29 (s, 1H, NH), 7.72 (d, *J *= 8.6 Hz, 1H, H_5_), 7.62 (d, *J *= 8.3 Hz, 2H, H_2›_, H_6›_), 7.52 (t, *J *= 8.3 Hz, 2H, H_3›_, H_5›_), 7.06-7.03 (m, 2H, H_6_, H_8_), 6.23 (s, 1H, H_3_), 4.86 (s, 2H, OCH_2_), 2.40 (s, 3H, CH_3_); ^13^C NMR (125 MHz, DMSO-*d*_6_): 166.0, 160.7, 160.0, 154.5, 153.3, 137.6, 131.6, 126.5, 121.6, 115.4, 113.6, 112.3, 111.4, 101.7, 67.2, 18.1; Anal. Calcd for C_18_H_14_BrNO_4_: C, 55.69; H, 3.63; N, 3.61. Found: C, 55.92; H, 3.45; N, 3.85.

*2-(2-Oxo-4-phenyl-2H-chromen-7-yloxy)-N-phenylacetamide (8i)*

Yield: 73 %; mp 230-232 ◦C; IR υ_max_ (KBr): 3351, 3133, 1716, 1680, 1621, 1540, 1401; ^1^H NMR (500 MHz, DMSO-*d*_6_): 10.19 (s, 1H, NH), 7.64 (d, *J *= 7.8 Hz, 2H, H_2›_, H_6›_), 7.56-7.53 (m, 5H, Ph), 7.39 (d, *J *= 8.9 Hz, 1H, H_5_), 7.33 (t, *J *= 7.8 Hz, 2H, H_3›_, H_5›_), 7.14 (d, *J *= 2.0 Hz, 1H, H_8_), 7.09 (t, *J *= 7.8 Hz, 1H, H_4›_), 7.03 (dd, *J *= 8.9, 2.0 Hz, 1H, H_6_), 6.26 (s, 1H, H_3_), 4.87 (s, 2H, OCH_2_); ^13^C NMR (125 MHz, DMSO-*d*_6_): 166.2, 161.4, 160.3, 155.7, 155.5, 1387, 135.4, 130.1, 129.3, 129.2, 128.8, 128.3, 124.2, 120.1, 113.2, 112.7, 112.1, 102.6, 67.7. Anal. Calcd for C_23_H_17_NO_4_: C, 74.38; H, 4.61; N, 3.77. Found: C, 74.08; H, 4.38; N, 3.92.

*2-(2-Oxo-4-phenyl-2H-chromen-7-yloxy)-N-(4-chlorophenyl)acetamide (8j)*

Yield: 75 %; mp 266-268 ◦C; IR υ_max_ (KBr): 3416, 1716, 1690, 1613, 1531, 1430; ^1^H NMR (500 MHz, DMSO-*d*_6_): 10.33 (s, 1H, NH), 7.67 (d, *J *= 8.8 Hz, 1H, H_5_), 7.57-7.53 (m, 5H, Ph), 7.40-7.38 (m, 4H, H_2›_, H_3›_, H_5›_, H_6›_), 7.14 (d, *J *= 2.4 Hz, 1H, H_8_), 7.03 (dd, *J *= 8.8, 2.4 Hz, H_6_), 6.27 (s, 1H, H_3_), 4.88 (s, 2H, OCH_2_); ^13^C NMR (125 MHz, DMSO-*d*_6_): 166.4, 161.4, 160.3, 160.3, 155.7, 155.5, 137.7, 135.4, 130.1, 129.3, 129.1, 128.9, 127.8, 121.6, 113.2, 112.8, 112.1, 102.6, 67.7; Anal. Calcd for C_23_H_16_ClNO_4_: C, 68.07; H, 3.97; N, 3.45. Found: C, 68.29; H, 4.16; N, 3.33.

*Antimicrobial activity*

Compounds 5a-h and 8a-j were evaluated for their antibacterial activity against Gram-positive (*Staphylococcus aureus *ATCC 6538p, *Staphylococcus epidermidis *ATCC 12228) and Gram-negative (*Escherichia coli *ATCC 8739, *Klebsiella pneumonia *ATCC 10031, and *Pseudomonas aeruginosa *ATCC 9027) bacteria using conventional agar-dilution method (32). The MIC (minimum inhibitory concentration) values were determined by comparison to ciprofloxacin as a reference drug. Two-fold dilution of the test compounds and the standard antibacterial agents were prepared in dimethyl sulfoxide (DMSO; 1 mL). Each dilute was added to molten Mueller-Hinton (MH) agar (19 mL) at 50 ◦C to give a final concentration of 100, 50, 25, 12.5, 6.25, 3.125, 1.56, 0.78, 0.39, 0.195, 0.098, 0.049, 0.025, 0.012, 0.006 and 0.003 μg mL^-1^. The bacterial inocula were prepared by suspending overnight colonies from MH agar media in 0.85% saline. The inocula were adjusted photometrically at 600 nm to a cell density equivalent to approximately 0.5 McFarland standards (1.5 × 108 CFU/mL). The suspensions were then diluted in 0.85% saline to make 107 CFU/mL. The plates were spot-inoculated with 1 μL of bacterial suspensions (104 CFU/spot); including a control plate containing 1 mL DMSO without any antibacterial agent. The plates were incubated at 35-37 °C and examined after 18 h. The MIC was determined as the lowest concentration of the agent that completely inhibits visible growth of the microorganisms.

*Antioxidant activity*

*DPPH radical scavenging assay*

Several concentrations of test compounds 5a-h and 8a-j in DMSO were prepared. The compound solution (1.0 mL) was added to the methanolic DPPH solution (2.0 mL, 0.1 mM), and the mixture was kept in the dark for 15 min. The absorbance at 517 nm was then measured by an UV ⁄ visible spectrophotometer. The percent scavenging activity was calculated using the following formula: inhibition (%) = 100. (Abs_control_) Abs_compound_) ⁄ Abs_control_. The DPPH radical scavenging activity of compounds was expressed in terms of IC_50_ (mM), which is obtained from linear regression plot between concentrations of test compound and percent inhibitions (33).

*Ferric reducing antioxidant power (FRAP) assay*

The FRAP assay reagent was prepared by adding 10 vol of 300 mM acetate buffer, pH 3.6 (3.1 g sodium acetate and 16 mL glacial acetic acid), 1 vol of 10 mM 2,4,6-tripyridyl-s-triazine prepared in 40 mM HCl, and 1 vol of 20 mM FeCl3. The mixture was diluted to 1/3 with methanol and prewarmed at 37 *◦C*. This reagent (3 mL) was mixed with 0.1 mL diluted test compounds 5a-h and 8a-j. The mixture was shaken and incubated at 37 *◦C *for 8 min, and the absorbance was read at 593 nm. A blank with only 0.1 mL methanol was used for calibration. The difference in absorbance between the tested sample and the blank reading was calculated, and the data were expressed as mM of ferric reduced to ferrous form (34).

## Conclusion

In summary, a series of new sulfonamide and amide derivatives containing coumarin derivatives were synthesized with a simple procedure in two steps starting from sulfanilamide and aniline derivatives using chloacetyl chloride and various coumarin derivatives. The biological activity of target compounds was screened. Although they did not exhibit good antimicrobial activities but they showed moderate to noticeable antioxidant activities. 
